# Diagnostic dilemmas in Intraductal papillomas of the breast - Experience at Sultan Qaboos University Hospital in the Sultanate of Oman

**DOI:** 10.12669/pjms.312.6476

**Published:** 2015

**Authors:** Adil Aljarrah, Kamran Ahmad Malik, Husam Jamil, Zoheb Jaffer, Sukhpal Sawhney, Ritu Lakhtakia

**Affiliations:** 1 Adil Aljarrah, Breast Unit, Department of Surgery, Sultan Qaboos University Hospital, Muscat, Sultanate of Oman; 2 Kamran Ahmad Malik, Breast Unit, Department of Surgery, Sultan Qaboos University Hospital, Muscat, Sultanate of Oman; 3 Husam Jamil, Breast Unit, Department of Surgery, Sultan Qaboos University Hospital, Muscat, Sultanate of Oman; 4 Zoheb Jaffer, Breast Unit, Department of Surgery, Sultan Qaboos University Hospital, Muscat, Sultanate of Oman; 5 Sukhpal Sawhney, Breast Unit, Department of Radiology, Sultan Qaboos University Hospital, Muscat, Sultanate of Oman; 6 Ritu Lakhtakia, Breast Unit, Department of Pathology, COM&HS, Sultan Qaboos University Hospital, Muscat, Sultanate of Oman

**Keywords:** Nipple Discharge, Breast Intraductal Papilloma, Duct Ectasia, Microdochectomy, Ductal Carcinoma In Situ (DCIS), Lobular Carcinoma In Situ LCIS

## Abstract

**Objectives:**

The aim of this retrospective study was to correlate the significance and accuracy of the colour of nipple discharge and breast ultrasound imaging in the diagnosis of intraductal papilloma.

**Methods:**

This is a retrospective study of 34 patients who underwent 36 microdochectomies in Sultan Qaboos University Hospital (SQUH) in the Sultanate of Oman, over a 4 year period of January 2009 till December 2012. The confounders considered were patient age, physical examination findings, nipple discharge cytology result, ultrasound results and biopsy report following microdochectomy. Comparisons analysis, charts and graphs were made using the SPSS software (version 20).

**Results:**

The mean age of the patients was 44(27-73) years old. Twenty-seven out 36 (75%) patients had presented with nipple discharge, 14 out 27 (52%) had blood stained nipple discharge and 13(48%) with coloured discharge (yellow, brown and green), 9 patients had no discharge. The final histopathology showed intraductal papilloma 13 (36%), duct ectasia 18(50%), DCIS 1 (2.7%), fibrocystic disease 3(8.3%) and LCIS 1(2.7%). Thirteen out of 36 had intraductal papilloma on final histopathology. The correlation between blood stained discharge and final histopathology of intraductal papilloma was insignificant (p=0.44).

**Conclusion:**

Nipple discharge is irrelevant to the diagnosis of intraductal papilloma. Spontaneous nipple discharge regardless of color is to be referred to breast surgeon and to be assessed with triple assessment. Surgery remains the mainstay of treatment.

## INTRODUCTION

There is a vast variety of clinical and radiologic manifestations of intraductal papillomatous lesions of the breast. Macroscopically, the lesion presents as an oval or roundish mass located within a dilated lactiferous duct; the mass may be pedunculated or broad-based, and it generally measures a few millimeters in diameter. Histological analysis reveals proliferation of the ductal epithelium surrounded by myoepithelial cells and a fibro vascular stroma.^1^ Although these lesions are benign, there is controversy about their diagnosis, mainly because of diversity in their clinical presentation and histopathology.^2^ Clinically, they present most commonly as nipple discharge, known as pathological nipple discharge, which can be either blood stained, serous or coloured.^3^ Blood stained or clear nipple discharge, usually of less than 6 months duration. The blood stained nipple discharge is thought to be due to twisting of papilloma on its fibro vascular pedicle leading to necrosis, ischemia and intraductal bleeding.^4^ Management is multidisciplinary, requiring triple assessment with detailed history and clinical examination, imaging with mammogram or Ultrasonography, cytology of the discharge and biopsies followed by surgical intervention.

Ultrasonography of papillary lesions typically shows a solid, oval, intraductal mass, often associated with duct dilatation. A cystic component is also commonly seen, and lesions may appear hyper vascular on color Doppler.^5^,^6^ MRI has been used as part of diagnostic imaging, but with high false positivity.^7^ Pathological nipple discharge is defined as spontaneous, persistent, unilateral and coming from a single duct during non-lactational period. The rate of malignancy is reported to be 3.1%.^8^

The aim of this retrospective study was to correlate the significance and accuracy of the colour of nipple discharge and breast ultrasound imaging in the diagnosis of intraductal papilloma.

## METHODS

We conducted a retrospective study of 34 patients who underwent 36 microdochectomies in Sultan Qaboos University Hospital (SQUH) in the Sultanate of Oman, over a 4 year period of January 2009 till December 2012.

Patient inclusion criteria were all female, all ages, breast ultrasound showing intraductal lesion, patient undergoing microdochectomies. Ultrasonography and histopathological correlation was determined before and after microdochectomies respectively. The confounders considered were patient age, physical examination findings, nipple discharge cytology, ultrasound results and biopsy report following microdochectomy.

All patients were treated at the breast Unit in SQUH and were operated by the same surgeon using same technique. Identification of the duct was by ultrasound guided skin marking and the insertion of a lacrimal probe in the opening of the duct with discharge at the time of induction. All patients underwent day care surgery under general anesthesia.

Ultrasonography was performed by a single radiologist on a Philips IU 22 machine using a linear array 12.5 MHz transducer. Patients were scanned in the supine and or contra-lateral oblique positions depending on the sites of the lesions. The findings were reported according to the American College of Radiology’s BIRADS sonographic classification.

### Fine needle aspiration cytology and nipple discharge

Smears from these specimens were stained with Papanicolaou, Haematoxylin and eosin and Diff Quik stains. Smears were examined for adequacy, morphologic characteristics of ductal epithelial cells (architecture and cytology), presence of myoepithelial cells, background stromal material and inflammatory cells.

### Biopsy

**Fig.1:**
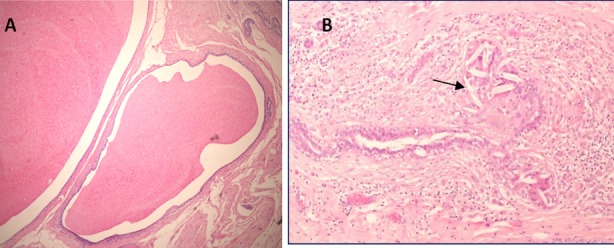
Duct ectasia: A. Two dilated ducts (D) distended by granular eosinophilic secretions (H&E ×40). B: A ruptured duct surrounded by a lympho-histiocytic inflammatory response and focal collections of cholesterol foreign body giant cells (arrow) (H&E ×100)

Specimens of microdochectomy were sampled for evaluation by measuring, orienting and by a gross description. Margins were inked. Specimens were serially sliced along the length of the ducts for complete evaluation. Papillomas were recorded as single or multiple and maximum dimension measured. On Haematoxylin and eosin sections, morphology of the ductal changes and papillary lesions was evaluated; categorisation and classification of benign and malignant lesions was made; where necessary immunohistochemical demonstration of myoepithelial cells with p63, CK5/6 and for receptor status (ER/PR expression) was performed. Besides the radiologically detected duct lesion, comment was made on changes in the terminal duct lobular (TDLU) units (Fig.1) in the surrounding breast including presence of DCIS or LCIS. In malignant lesions, comment on the status of the inked margins was made for adequacy of excision.

### Statistical Analysis

Comparisons analysis, charts and graphs were made using the SPSS software (version 20).

## RESULTS

A total of 34 patients presented to the breast clinic at SQUH; all patients had tripple assessment with history and clinical examination, breast imaging with ultrasound or mammogram and breast ultrasound according to patients age. All 34 patients had a breast ultrasound; only 26 patients had a mammogram. Thirty-four patients underwent 36 years microdochectomies, as 2 patients had bilateral surgery. The mean age of the patients was 44(27-73) years. 27(75%) patients out of 36 had nipple discharge symptoms; 14(39%) out of 36 had blood stained nipple discharge and 13(36%) with coloured discharged varied between green, brown and yellow; 9 patients had no discharge but clinically had palpable lumps in the retro areolar region which on ultrasound showed an intraductal lesion. Ultrasound in all patients was suggestive of intraductal papilloma, and reported as BIRADS III. The side of the lesion was 19(53%) left breast and 17(47%) right breast. The specificity of Ultrasound showed 13(36%) when was correlated to final histopathology.

The final histopathology showed, benign intraductal papilloma 13(36%), duct ectasia 18(50%), DCIS 1 (2.7%), fibrocystic disease 3(8.3%) and LCIS 1(2.7%). The correlation between the type of discharge and the final histopathology of intraductal papilloma is shown in Table-I. P value was insignificant (P Value=0.44). One (2.7%) out of 36 patients showed DCIS, One (2.7%) out of 36 patients showed LCIS, and 50% had duct ectasia, (Table-II).

**Table-I:** Correlation between color of discharge and final histopathology.

Clinical presentations	Histopathology		
	Papillomas	Non Papillomas	Total
Blood stained discharge	4(29%)	10(71%)	14
Coloured discharge	6(46%)	7(54%)	13
Total	10	17	27

**Table-II:** Clinical Presentation and final histopathology Final Histopathology diagnosis.

Clinical Presentation	DCIS	Papilloma	Ductal ectasia	Fibrocystic	LCIS	Total
Blood stained discharge	1 (7%)	4 (29%)	7 (50%)	1 (7%)	1 (7%)	14
Coloured discharge	0	6 (46%)	6 (46%)	1 (8%)	0	13
Reteroareolar mass	0	3 (33%)	5 (56%)	1 (11%)	0	9

## DISCUSSION

In this study our results showed that the colour of nipple discharge is irrelevant to the diagnosis of intraductal papilloma, in that only 29% of patients with intraductal papilloma presented with blood stained discharge, whereas 46% of those patients presented with colored discharge. This result is similar to other studies.^4^

There is currently no imaging technique that constitutes the gold standard for a definitive diagnosis of intraductal papillomas. Ultrasonography was used in our study by which some cases were found to have multiple intra-ductal papilloma’s within the same dilated duct and others had solitary papillomas. Ultrasound showed specificity of 36% for intraductal papilloma, when correlated with final histopathology, which was not similar to other studies, this may be because ultrasound is very operator dependent.^9^ MRI and ductoscopy have been used, but have been used in limited center.

Microdochectomy remains an effective surgical approach for management of intraductal papilloma. Surgical excision is to be performed once diagnosis has been made FNAC or core biopsy: it allows thorough assessment of even the smallest lesions.^10^ Intraductal papilloma can be multiple or single and occasionally may develop malignancy. In our study co-existent DCIS was present in 2.7% of patients and LCIS was also 2.7%, similar to other studies.^6^

**Fig.2:**
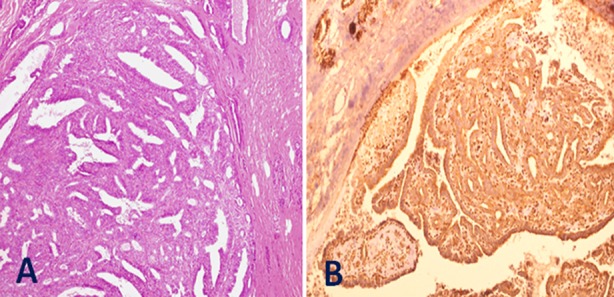
A: The large duct shows papillae with fibrovascular cores with no evidence of invasion (H&E ×100). B: The myoepithelial cell lining is preserved as demonstrated by p63 nuclear staining (Inset: DAB ×100)

All our specimen were evaluated on hematoxylin and eosin sections and immunohistochemical characterization used where applicable, which gave us more accurate results (Fig. 2a and 2b).

**Fig.3:**
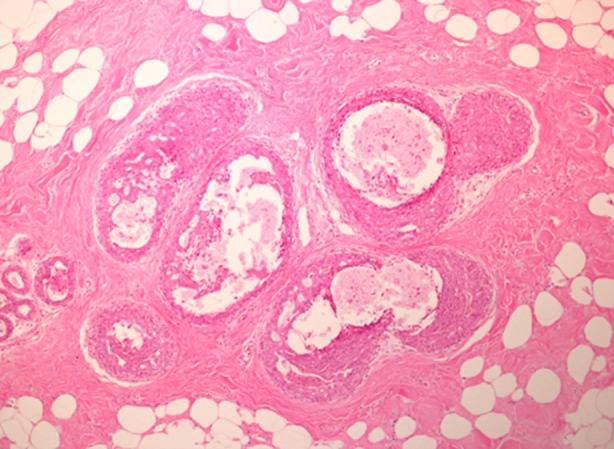
Intraductal papilloma with Ductal Carcinoma In Situ (DCIS).

**Fig.4:**
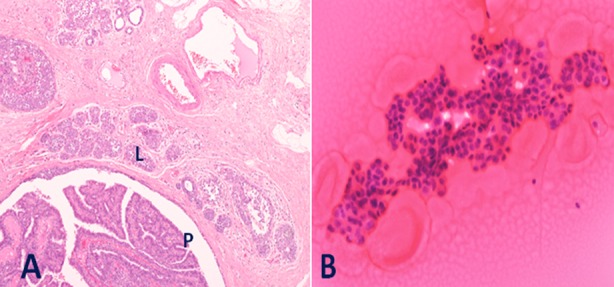
A. Intraductal papilloma with Lobular Carcinoma In Situ (LCIS): A large duct is distended by a benign papillary growth with fibrovascular cores (P). Adjoining lobules show LCIS with enlarged acini filled with monomorphic cells with minimal atypia (L). (H&E ×100). B: Fine needle aspirate had revealed a cellular papillary lesion (H&E ×400).

Multiple papillomas have similar features but ductal epithelial cells are more frequently associated with hyperplasia, atypia, DCIS (Fig.3) or LCIS (Fig. 4a and 4b), sclerosing adenosis or a radial scar.^11^

**Fig.5:**
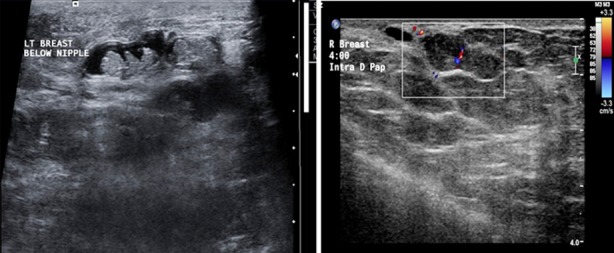
A: Multiple intra-ductal papilloma’s within the same dilated duct. B: A papilloma arising from the wall of the duct nearly filling the lumen of the duct. A vascular stalk is seen.

**Fig.6:**
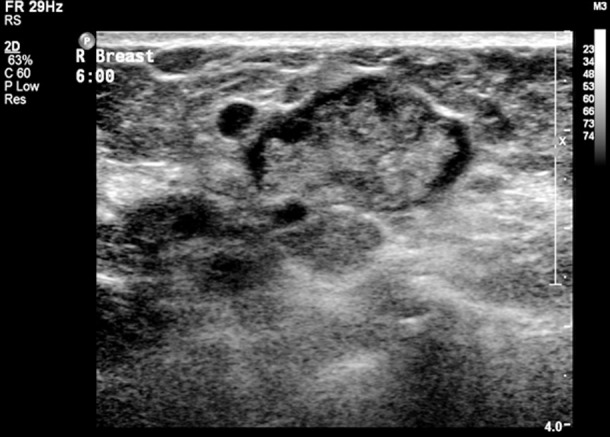
A papillary growth arising from the walls of a peripheral duct with a narrow base of attachment.

We conclude that all spontaneous nipple discharge regardless of their color be referred to a breast surgeon for triple assessment. Ultrasound has been used conventionally for imaging these patients, however alternative imaging techniques may be needed for greater accuracy in diagnosing intraductal papilloma. Surgery remains the mainstay of treatment, however further study needs to be carried out to know more diagnostic imaging and potential for malignant changes in intraductal papilloma.
